# Ethnic differences in plasma aldosterone concentration and regulatory mechanisms of aldosterone: protocol for a systematic review and meta-analysis

**DOI:** 10.1136/bmjopen-2025-105167

**Published:** 2025-10-15

**Authors:** Aditya Sharma, Gisele Bentley, Spoorthy Kulkarni, Ian Wilkinson

**Affiliations:** 1 Department of Medicine, University of Cambridge, Cambridge, UK

**Keywords:** Hypertension, CLINICAL PHARMACOLOGY, General endocrinology

## Abstract

**Abstract:**

**Introduction:**

Black ethnic cohorts, when compared with white cohorts, have disproportionately higher rates of essential hypertension and related complications. Ethnic differences in the renin-angiotensin-aldosterone system have been identified in black ethnic cohorts displaying a low-renin, salt-sensitive phenotype in comparison to white individuals. Studies have highlighted lower levels of aldosterone in black cohorts compared with white cohorts. However, when renin is considered, in the form of the aldosterone-renin ratio (ARR), the ARR is higher in black cohorts. The Framingham study highlighted that people in the upper quartile for baseline aldosterone were more likely to have a higher blood pressure and develop hypertension at 4 year follow-up. Therefore, the inappropriately suppressed aldosterone may be contributing to the ethnic differences in hypertension prevalence and prognosis.

**Methods and analysis:**

Four databases will be searched (MEDLINE, Embase, Scopus and Cochrane Library) to identify eligible studies from database creation to August 2025. Two investigators will independently review the search results and document reasons for full-text exclusions. The main outcomes are to assess if there are ethnic differences in baseline aldosterone, baseline plasma renin activity (PRA) and ARR. We will also look at ethnic differences in regulatory mechanisms of aldosterone, such as serum potassium and 24 hour urinary electrolytes, that may explain the potential differences in aldosterone and ARR in black and white ethnic cohorts. Studies looking at normotensive and hypertensive individuals will be included. Studies in paediatric populations (<18 years) will be excluded. We will include studies without language restriction. Risk of bias assessment will be completed with a modified Newcastle-Ottawa scale. Subgroup analysis and sensitivity analyses will be completed to verify the accuracy of the study results and the consistency of the inferences.

**Ethics and dissemination:**

As this review will involve analysis of previously published data, ethical approval is not required. The results will be submitted to a peer-reviewed journal.

**PROSPERO registration number:**

CRD420251025642.

**Strengths and limitations of this study**

- Investigating ethnic differences in aldosterone may provide insights into ethnic differences in prevalence and severity of hypertension.
- Two reviewers with experience of systematic review will independently screen, extract data, analyse data and assess risk of bias across eligible studies.
- There are many factors that affect baseline aldosterone, which will result in significant heterogeneity.
- Assays for aldosterone have changed over time, limiting potential comparisons.

## Introduction

 Black ethnic cohorts, when compared with white cohorts, have disproportionately higher rates of essential hypertension^1^ and its complications, including stroke, end-stage renal failure and hypertension-mediated end-organ damage.^2 3^ This discrepancy may be partially explained by differences in the pathophysiology of the disease. Extracellular fluid volume is an important contributor to the pathogenesis of hypertension in black people.^4 5^ The renin-angiotensin-aldosterone-system (RAAS) maintains extracellular volume in the setting of salt or volume loss through vasoconstriction and salt retention.^6^ Black cohorts are more likely to be salt sensitive,^7^,^9^ which is defined as people who display an exaggerated elevation in blood pressure in response to dietary sodium.^10^ The suppression of renin and the RAAS pathway leads to black cohorts displaying a low-renin, salt-sensitive phenotype of hypertension.

Higher plasma aldosterone concentration (PAC) is linked with the development of hypertension. The Framingham Offspring study demonstrated that people with PAC in the upper quartile of the normal range were more likely to have higher BP and be hypertensive at 4 year follow-up compared with people in the lowest quartile of aldosterone.^11^ PAC has also been shown to be positively related to BP in black cohorts across multiple studies.^12^,^15^

Despite this evidence, PAC is lower in black cohorts compared with whites, in normotensive,^16^ hypertensive^16 17^ and paediatric populations.^14 18^ Some studies highlight the importance of interpretation of PAC with respect to renin.^19 20^ While the absolute PAC may be low, aldosterone may be in excess relative to the plasma renin activity (PRA). This relative aldosterone excess can be highlighted through a high aldosterone-renin ratio (ARR). The ARR has been found to be higher in black participants in multiple studies, when compared with white participants.^21 22^

The role of RAAS, in particular aldosterone, in the pathogenesis of hypertension in black cohorts has yet to be fully elucidated. The combination of low PAC and a more positive sodium balance may suggest a diminished role of aldosterone in determining BP in black cohorts. Alternatively, the higher ARR in black cohorts demonstrates that aldosterone may have a larger part to play in the pathogenesis of hypertension in black populations; this could also contribute to the elevated sodium balance that is seen.

Ethnic differences in the regulation of aldosterone may account for differing values in normotensive and hypertensive black and white cohorts. Aldosterone production in vitro is affected by more than 20 hormones, autacoids, ions and nutrients.^23 24^ It is probable, therefore, that regulation of aldosterone secretion in humans is more complex than classical schemes that involve primarily angiotensin II and potassium. Moreover, plasma levels of aldosterone are not always predictable from the PRA and the concentration of potassium.

Understanding whether there are ethnic differences in aldosterone regulation, and what the factors are, is crucial for providing relevant information and care for patients. For example, the prevalence of hypertension in black people may be reduced if reduced dietary potassium is identified to be a factor explaining the pathogenesis of inadequately suppressed aldosterone. Furthermore, preferential prescription of mineralocorticoid receptor antagonists (MRA) in black patients with hypertension may help address the raised ARR seen in the low-renin black cohorts. Finally, drug development targeting aldosterone is a rapidly expanding field,^25^ and so, identifying populations that may benefit the most will be useful for clinicians and patients.

Significant variation exists in baseline aldosterone due to the sensitivity of the hormone to changes in the environment. These include, but are not limited to, time of day, standing or supine position, diet and fluid intake. Moreover, the assays used to measure aldosterone are significantly variable in meta-analysis,^26^ as highlighted by the Endocrine Society.^27^ It is clear, therefore, that measurement of aldosterone poses significant challenges. By identifying ethnic differences, we may provide useful information for the creation of more appropriate cut-offs and normal ranges.

### Objectives

1. To investigate the degree of corroboration between studies on the baseline levels of PAC between white and black cohorts.
2. To investigate whether PAC is inadequately suppressed, relative to renin, in black people compared with white people, using the ARR.
3. To investigate hypothesised factors contributing to ethnic differences in PAC regulation.

## Methods and analysis

This systematic review and meta-analysis was registered on the International Prospective Register of Systematic Reviews (CRD420251025642). This study protocol is reported in accordance with the Preferred Reporting Items for Systematic Review and Meta-Analysis Protocols guidelines to ensure transparency and completeness in reporting.^28^

### Data sources and search strategy

**Figure 1:**
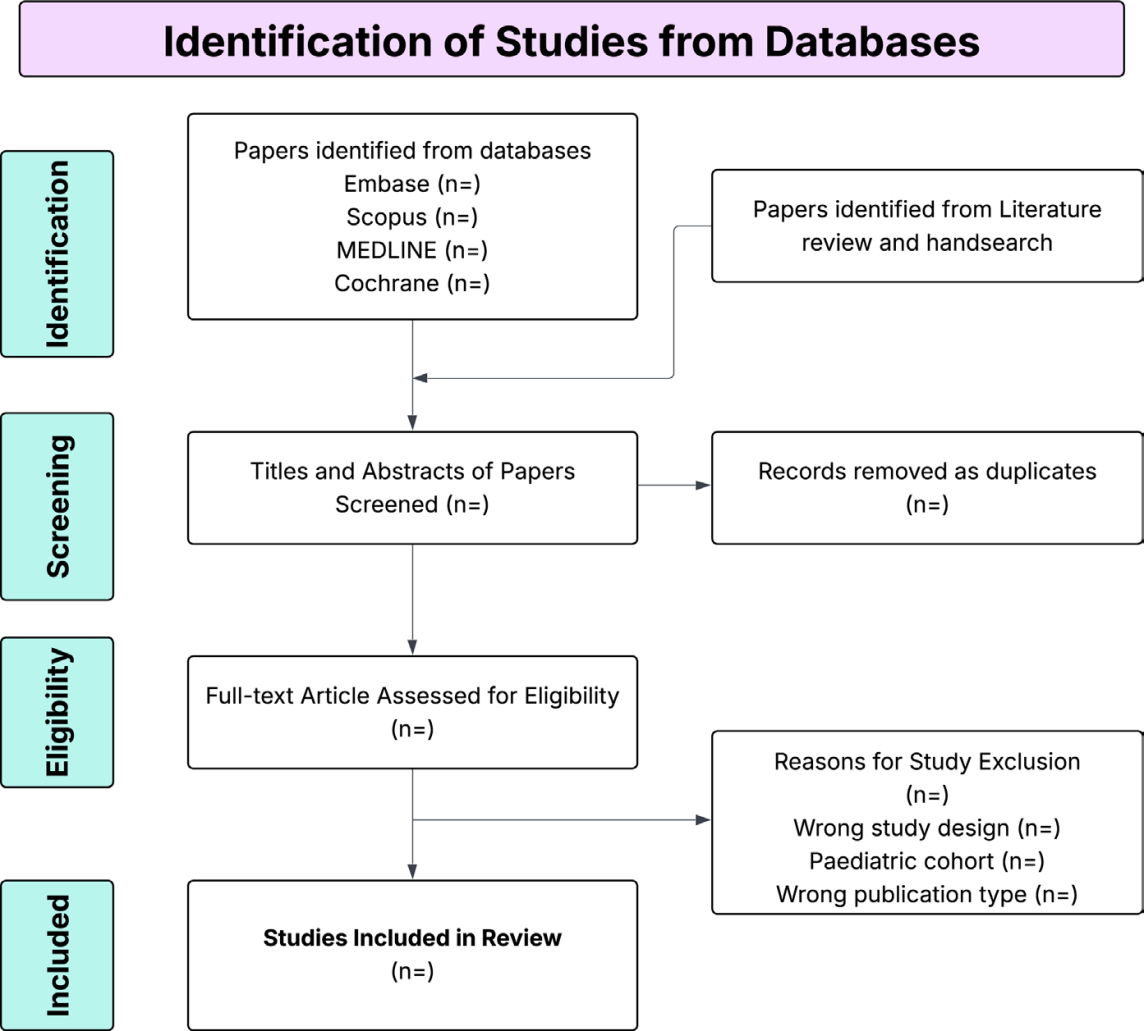
The Preferred Reporting Items for Systematic Review and Meta-Analysis (PRISMA) flow diagram that will be used for literature screening.

A comprehensive search will be performed independently by two reviewers. Differences will be resolved with discussion and consultation with a third reviewer, if required. Four databases will be searched from date of creation to date of search (Embase, MEDLINE, Cochrane Library, Scopus). Manual searches of references from identified articles and previous systematic reviews will also be conducted to identify any more relevant studies. The full flow chart is included in figure 1. No language restrictions will be applied during the search process. Studies published in languages not known to the authors will be translated into English using Google Translate to assess eligibility, extract data and incorporate their findings into the review.

The detailed search strings for each database are presented in online supplemental table S1. Searches will be based on these keywords: (1) aldosterone and (2) ethnicity. Use of associated terms and alternative names, like ‘white’ and ‘Caucasian’, will increase the chance of finding all relevant papers. Moreover, by keeping the searches broad, we hope to find all relevant papers.

### Eligibility criteria

1. Adult (≥18 years) men and women. Papers including normotensive individuals will be included. Studies looking at hypertensive individuals will be included if the participants are not currently prescribed anti-hypertensive treatment or have had at least 1 week washout from anti-hypertensive medication.
2. The comparison is between ethnic groups; therefore, included studies must provide data grouped by ethnicity, including black and white ethnic cohorts.
3. Studies that documented baseline plasma aldosterone concentration measured with standardised units, including but not limited to, pg/mL, ng/dL, pmol/L.
4. We considered cohort, cross-sectional and case-control studies, as well as primary or secondary analyses of randomised controlled trials.

### Exclusion criteria

1. Studies in children and adolescents. Studies with participants who have evidence of end-organ damage or participants with an identified cause of hypertension (secondary hypertension). Studies including individuals on BP-altering medication. Studies where PAC is measured on a controlled diet of sodium or potassium.
2. Studies where baseline plasma aldosterone concentration is not measured in black and white ethnic cohorts or where a measure of variance is not included. Studies with a sample size <5 people per ethnic group will be excluded.
3. Studies in animals, case studies, case series, systematic reviews, meta-analyses, narrative reviews, editorials and conference abstracts will be excluded.

### Study selection

Search results from the databases will be uploaded into Covidence, a web-based software for systematic reviews. This software will automatically remove duplicates identified across the databases (Covidence systematic review software, Veritas Health Innovation, Melbourne, Australia).

Two independent reviewers will review the papers returned from the searches for eligible studies. Initial screening will be based on title, abstract and keywords. Ineligible studies will be excluded from further review.

Full texts of the remaining studies will be reviewed to assess their eligibility based on the inclusion and exclusion criteria. Any disagreements will be resolved with the consultation of a third reviewer. Reasons for full text paper exclusion will be documented and supplied in the online supplemental materials.

### Data extraction

A pre-designed data extraction table will be used for collating the data from eligible papers. Relevant information will include: title, authors, year of publication, journal, country of publication, study design, total number of participants, number of black and white participants, whether population is normotensive/hypertensive/mixed, sex of participants, protocol for PAC collection measurement, body mass index (BMI), serum sodium and potassium, 24 hour urinary sodium and potassium, PAC, measures of renin including PRA and direct renin concentration, aldosterone-renin-ratio (ARR), systolic blood pressure (SBP) and diastolic blood pressure (DBP). Primary and secondary outcomes of the studies will be collected as well. Units for PAC, measures of renin and ARR will be collected too. Values, when presented in figure form, will be extracted using publicly available software (WebPlotDigitizer; https://automeris.io/WebPlotDigitizer).

Aldosterone has different standardised units, the most common reported in the literature are ng/dL, pmol/L and pg/mL. Conversion from pmol/L into ng/dL will be done using a standardised formula.^29^

As we are looking at baseline aldosterone, we are expecting to find aldosterone reported as mean and SD.^30^ However, aldosterone is often reported as median and IQR to account for the skewed distribution. Previous studies have reported aldosterone values as geometric mean and 95% CIs. To calculate a mean difference, we will convert geometric mean and median into arithmetic mean. The formulas used for these conversions will account for the non-normal distribution of aldosterone and assume log distribution. The R code that will be used is listed in the online supplemental material.

### Quality assessment

Two reviewers will independently evaluate the Risk of Bias (RoB) of the included studies using a modified version of the Newcastle-Ottawa scale,^31^ with disagreements resolved with a third reviewer. As most eligible studies were cross-sectional or cohort studies, we will apply the scale to all studies. The following criteria will be assessed: representativeness of ethnic cohorts; sample size ascertain; exposure ascertainment; adequate baseline; comparability; assessment of outcome quality measurement. Online supplemental table S2 highlights the criteria that will be used for the assessment. Quality assessment scores of 0–3, 4–6 and 7–9 will represent high, uncertain and low RoB studies, respectively.

### Pairwise meta-analysis

Analysis of the data will be conducted in R Statistical Software (V.4.4.3; R Core Team 2021), using the ‘meta’ package. Given the variability of hormones like aldosterone, the DerSimonian-Laird random-effects model will be used in traditional pairwise meta-analysis.^32^ Standardised mean difference using Hedges’ g and 95% CIs will be compared across studies. We will report the absolute mean difference in the supplementary data as the difference may provide more clinically relevant information. The *I*^2^ statistic will be used to measure the between-study heterogeneity. Based on the availability of reported data, meta-regression analyses will be conducted to see if heterogeneity of effect size (ethnic difference in baseline aldosterone) can be explained by measured variables such as serum potassium and 24-hour urinary potassium. Based on collected data, analysis of plasma aldosterone concentration, PRA and ARR will be conducted. Subgroup analysis will look at normotensive and hypertensive cohorts to see if there are differences in aldosterone. Furthermore, papers that report ethnic differences in men and women will also undergo subgroup analysis to assess for sex differences in aldosterone across ethnicities.

### Sensitivity analysis

The Cochrane Handbook states that heterogeneity should be considered significant if the *I²* statistic is 50% or greater, combined with a *p*<0.05.^33^ Sensitivity analyses including checking for publication bias, assessing for outliers and influential studies and subgroup analysis will be conducted. Publication bias will be tested by a funnel plot and Egger’s regression asymmetry test.^34^ Trim and fill analysis will be used to look for missing studies.^35^

### Patient and public involvement

Patients and/or the public were not involved in this study.

## Ethics and dissemination

This protocol includes only published literature and there is no involvement of patients or participants; therefore, no ethics approval is necessary. The results will be submitted to a peer-reviewed journal.

## Supplementary material

10.1136/bmjopen-2025-105167 online supplemental file 1
