# Supplementary material for: Ethnic differences in plasma aldosterone concentration and regulatory mechanisms of aldosterone: protocol for a systematic review and meta-analysis
Source: BMJ Open. 2025 Oct 15;15(10):e105167. doi: 10.1136/bmjopen-2025-105167 (PMC12530402; doi:10.1136/bmjopen-2025-105167)
Supplement: online supplemental file 1 [file bmjopen-15-10-s001.docx]

**Table S1.** Search strategies for (A) MEDLINE, (B) Embase, (C) Scopus, and (D) Cochrane CENTRAL without language restriction from database inception to date of search.

**(A) MEDLINE (1946-2025)**

| **#** | **Searches** | **Results** | **Search Type** |
| --- | --- | --- | --- |
| 1 | aldosterone.mp. or aldosterone release/ or exp aldosterone/ or aldosterone blood level/ |  | Advanced |
| 2 | renin angiotensin system.mp. or exp renin angiotensin aldosterone system/ |  | Advanced |
| 3 | ethnic difference/ or ethnic*.mp. |  | Advanced |
| 4 | race.mp. or exp race/ or race difference/ |  | Advanced |
| 5 | white.mp. or Caucasian/ |  | Advanced |
| 6 | black.mp. or Black person/ or african.mp. or African/ |  | Advanced |
| 7 | 1 or 2 |  | Advanced |
| 8 | 3 or 4 or 5 or 6 |  | Advanced |
| 9 | 7 and 8 |  | Advanced |

**(B) Embase (1974-2025)**

| **#** | **Searches** | **Results** | **Search Type** |
| --- | --- | --- | --- |
| 1 | aldosterone.mp. or aldosterone release/ or exp aldosterone/ or aldosterone blood level/ |  | Advanced |
| 2 | renin angiotensin system.mp. or exp renin angiotensin aldosterone system/ |  | Advanced |
| 3 | ethnic difference/ or ethnic*.mp. |  | Advanced |
| 4 | race.mp. or exp race/ or race difference/ |  | Advanced |
| 5 | white.mp. or Caucasian/ |  | Advanced |
| 6 | black.mp. or Black person/ or african.mp. or African/ |  | Advanced |
| 7 | 1 or 2 |  | Advanced |
| 8 | 3 or 4 or 5 or 6 |  | Advanced |
| 9 | 7 and 8 |  | Advanced |

**(C) Scopus (1966-2025)**

TITLE-ABS-KEY((aldosterone OR renin AND angiotensin AND system) AND (ethnic* OR race OR black OR white))

**(D) Cochrane Library (1996-2025)**

((aldosterone OR renin?angiotensin system) AND (ethnic* OR race OR black OR white OR African OR caucasian))

| **Table S2.** Newcastle-Ottawa Scale for risk of bias of the studies that will be included in the systematic review | | | | | | |
| --- | --- | --- | --- | --- | --- | --- |
|  | **Selection** | | | | **Comparability** | **Outcome** |
|  | Representativeness of ethnic cohorts | Sample Size | Ascertainment of exposure | Adequate baseline prior to intervention |  | Assessment of outcome |
| Points awarded: | General population-based study | Justified and satisfactory – sample size calculation provided | Detailed report on the methodology of aldosterone measurement. What time of day was it measured, were participants fasted, were they supine/standing/seated, how long were they supine/standing/seated, how many measurements were taken, description of assay used? | Detailed information regarding factors that may affect baseline aldosterone: how long was the washout from anti-hypertensives, were patients that were taking medication that may affect BP included in the analysis, were patients on a controlled salt diet included? | Two points awarded if aldosterone is reported as mean ± SD and in ng/dL.  One point awarded if aldosterone is given as mean ± SEM and in ng/dL.  One point awarded if aldosterone is reported as mean ± SD/SEM and unit is pmol/L or pg/mL | One point awarded for each criterion:  1. accredited aldosterone assay used  2. supine or seated measurement  3. statistical test information provided |
| Points not awarded: | Random sample or selected sample | Unsatisfactory | Incomplete methodology  No methodology reported | No information reported | No points awarded if aldosterone is given as geometric mean and 95% confidence intervals or median and IQR.  No points awarded if aldosterone is given in graphical form, | No information provided |

# R code for geometric mean and 95% CI to arithmetic mean and SD

GM <-

CI_lower <-

CI_upper <-

n <-

# Log-transform

log_GM <- log(GM)

log_L <- log(CI_lower)

log_U <- log(CI_upper)

# Calculate sigma_log

sigma_log <- (log_U - log_L) / (2 * qnorm(0.975))

# Arithmetic mean and SD

AM <- exp(log_GM + sigma_log^2 / 2)

SD <- AM * sqrt(exp(sigma_log^2) - 1)

list(AM = AM, SD = SD, sigma_log = sigma_log)

# R code for lognormal estimation from median and IQR

median <-

q1 <-

q3 <-

# Estimate mu_log and sigma_log

mu_log <- log(median)

iqr_log <- log(q3) - log(q1)

sigma_log <- iqr_log / 1.35

# Step 2: Calculate mean and SD

mean_est <- exp(mu_log + (sigma_log^2)/2)

sd_est <- mean_est * sqrt(exp(sigma_log^2) - 1)

# Results

cat("Mean:", mean_est, "\nSD:", sd_est)
