# Supplementary material for: Ethnic differences in plasma aldosterone concentration and regulatory mechanisms of aldosterone: protocol for a systematic review and meta-analysis
Source: BMJ Open. 2025 Oct 15;15(10):e105167. doi: 10.1136/bmjopen-2025-105167 (PMC12530402; doi:10.1136/bmjopen-2025-105167)
Supplement: Supplementary file 2 [file bmjopen-2025-105167.reviewer_comments.pdf]

PEER REVIEW HISTORY

BMJ Open publishes all reviews undertaken for accepted manuscripts. Reviewers are asked to complete a checklist review form and are provided with free text boxes to elaborate on their assessment. These free text comments are reproduced below.

ARTICLE DETAILS

Title (Provisional)

Ethnic differences in plasma aldosterone concentration and regulatory mechanisms of aldosterone: protocol for a systematic review and meta-analysis

Authors

Sharma, Aditya; Bentley, Gisele; Kulkarni, Spoorthy; Wilkinson, Ian

---

VERSION 1 - REVIEW

**Reviewer**	**1**
**Name**	**Kyriakoulis, Konstantinos G**
**Affiliation**	**National and Kapodistrian University of Athens**
**Date**	**27-Jul-2025**
**COI**	**None**

This is the protocol of a meta-analysis planning to investigate the ethnic differences in plasma aldosterone concentration and respective regulatory mechanisms. Below, attached some comments:

1. Abstract: "The main outcomes are to assess if there are ethnic differences in baseline aldosterone, baseline plasma renin activity, and ARR" – what about studies with direct renin concentration?

2. Abstract: "We will also look at ethnic differences in regulatory mechanisms of aldosterone…" – such as? Clarifications would be helpful.

3. Introduction: "highlighted by the Endocrine Society. Endocrine society" – check for typos.

4. Methods and Analysis: will heterogeneity be assessed using meta-regression analyses? Will baseline treatment be taken into consideration? Studies will be included only if patients are untreated during baseline? Will studies with controlled salt/potassium diets be excluded?

5. Methods and Analysis/PRISMA flow diagram: what about citation handsearch of relevant papers/reviews/etc?

6. Methods and Analysis/Quality Assessment: better use the term Risk of Bias (RoB) and especially when you refer to “poor, fair, and good studies” better use “high, uncertain, low RoB”.

7. General Comment: is this a pairwise meta-analysis of aggregate data or a network meta-analysis? Somewhere the word “network” is used and relevant clarifications are needed.

VERSION 1 - AUTHOR RESPONSE

Response to reviewer’s comments:
1. studies looking at direct renin concentration (DRC) will be included in the review. This is outlined in the main paper – Data extraction in the methods section (page 5). The primary outcome is looking at studies that use plasma aldosterone concentration divided by plasma renin activity to calculate ARR. Therefore, DRC is not mentioned in the abstract section of the paper.
2. agreed. We have included serum potassium and 24-hour urinary electrolytes as an example of a commonly measured regulatory factor of aldosterone
3. removal of duplicate ‘Endocrine society’
4. thank you for the feedback. Provided studies report the relevant data, meta-regressions will be included in the analysis. Exclusion criterion 1 (page 4) states that only studies including untreated participants will be included. Exclusion of studies on sodium and potassium restricted diets is already listed in the exclusion criterion 1 (page 4)
5. thank you for the feedback. Included handsearch and literature reviews as methods of paper identification to the PRISMA flow chart. Manual search is already included in the ‘Data Sources and Search Strategy’ section in the main paper methods section (page 4)
6. Thank you for the feedback. Use of ‘high, low, and uncertain RoB’ is used in place of ‘poor, fair, and good RoB’
7. The descriptor ‘network’ has been removed as this is a pairwise meta-analysis of aggregate data

Once again, we would like to thank you both for your helpful comments. We hope that we have addressed these issues adequately and that you will find our revised manuscript suitable for publication.

VERSION 2 - REVIEW

**Reviewer**	**1**
**Name**	**Kyriakoulis, Konstantinos G**
**Affiliation**	**National and Kapodistrian University of Athens**
**Date**	**16-Sep-2025**

COI

Since this systematic review and meta-analysis will assess renin, aldo, and ARR, will also primary aldosteronism issues (e.g., positive PA screening prevalence between different ethnic groups) be assessed?

VERSION 2 - AUTHOR RESPONSE

Thank you for this comment. We are looking at aldosterone and ARR in essential hypertension. One of our exclusion criteria is studies including participants with secondary causes of hypertension, such as primary aldosteronism (PA).
Moreover, it is likely that we will not have original patient data for all included studies. Therefore, it will not be possible to calculate the proportion of each ethnicity that tests positive for PA. We will mention this in the limitations section of the full meta-analysis paper.
